# Dihydroartemisinin selectively inhibits PDGFRα-positive ovarian cancer growth and metastasis through inducing degradation of PDGFRα protein

**DOI:** 10.1038/celldisc.2017.42

**Published:** 2017-11-21

**Authors:** Xiaoguang Li, Qian Ba, Yanling Liu, Qingxi Yue, Peizhan Chen, Jingquan Li, Haibing Zhang, Hao Ying, Qiurong Ding, Haiyun Song, Hong Liu, Ruiwen Zhang, Hui Wang

**Affiliations:** 1 School of Public health, Shanghai Jiao Tong University School of Medicine, Shanghai, China; 2 Key Laboratory of Food Safety Research, Institute for Nutritional Sciences, Shanghai Institutes for Biological Sciences, Chinese Academy of Sciences, Shanghai, China; 3 Key Laboratory of Receptor Research, Shanghai Institute of Materia Medica, Chinese Academy of Sciences, Shanghai, China; 4 Department of Pharmacological and Pharmaceutical Sciences, College of Pharmacy, University of Houston, Houston, TX, USA

**Keywords:** Dihydroartemisinin, Epithelial–mesenchymal transition, metastasis, ovarian cancer, PDGFRα

## Abstract

To develop traditional medicines as modern pharmacotherapies, understanding their molecular mechanisms of action can be very helpful. We have recently reported that Artemisinin and its derivatives, which are clinically used anti-malarial drugs, have significant effects against ovarian cancer, but the direct molecular targets and related combination therapy have been unclear. Herein, we report that dihydroartemisinin, one of the most active derivatives of Artemisinin, directly targets platelet-derived growth factor receptor-alpha (PDGFRα) to inhibit ovarian cancer cell growth and metastasis. Dihydroartemisinin directly binds to the intercellular domain of PDGFRα, reducing its protein stability by accelerating its ubiquitin-mediated degradation, which further inactivates downstream phosphoinositide 3-Kinase and mitogen-activated protein kinase pathways and subsequently represses epithelial–mesenchymal transition, inhibiting cell growth and metastasis of PDGFRα-positive ovarian cancer *in vitro* and *in vivo*. A combinational treatment reveals that dihydroartemisinin sensitizes ovarian cancer cells to PDGFR inhibitors. Our clinical study also finds that PDGFRα is overexpressed and positively correlated with high grade and metastasis in human ovarian cancer. Considering that Artemisinin compounds are currently clinically used drugs with favorable safety profiles, the results from this study will potentiate their use in combination with clinically used PDGFRα inhibitors, leading to maximal therapeutic efficacy with minimal adverse effects in PDGFRα-positive cancer patients. These findings also shed high light on future development of novel Artemisinin-based targeted therapy.

## Introduction

Ovarian cancer poses a major health threat as the sixth leading cause of cancer-related death in women in developed countries and the eighth in developing countries [1]. Clinically, it is the most lethal gynecological malignancy, which is considered to be due to the advanced stage of the disease at the time of diagnosis, its highly metastatic nature, and the lack of effective treatments for advanced disease [2]. Therefore, the development of novel, effective and safe therapeutic agents by ensuring a careful alignment of the target, drug, patient and regimen design can lead to a better outcome for ovarian cancer patients [3, 4].

Considerable efforts have been devoted to evaluating several classes of conventional chemotherapeutic agents for ovarian cancer therapy, such as paclitaxel and platinum-based agents. However, clinical studies indicate that the clinical response rates to these treatments are low, and the clinical improvement is marginal after therapy, especially in patients with advanced disease (stages III–IV), which may be due to the late diagnosis, persistent disease dormancy and drug resistance [3, 5, 6]. It is hoped that basic and translational research aimed at the development of targeted therapy for ovarian cancer can identify etiological factors for patients with heterogeneous ovarian cancers, as well as predictive biomarkers for a better histopathological and molecular diagnosis and for the selection of an optimal therapeutic regimen, which will provide a basis for individualized targeted therapy and the discovery and development of more effective therapeutic agents [2, 5, 7].

Artemisinin (ART) and its derivatives, which are clinically used as anti-malarial agents worldwide, are regarded as one of the greatest recent clinical successes arising from traditional medicine [8–11]. Recently, ART derivatives have been shown to have anticancer properties, with low host toxicity [11–13]. One of the main reasons for developing ART and its analogs for cancer therapy is the safety profile of this class of compounds. ART derivatives such as dihydroartemisinin (DHA) and artesunate were found to be active against a variety of tumor cell lines including colon, breast, liver, lung, pancreatic cancer and sensitized cancer cells to conventional chemotherapy [14–17]. Additionally, human studies of individual cases [18, 19] and clinical trials support ART derivatives as a primary or adjunct antitumor intervention, particular for lung cancer, colon cancer and prostate cancer [20–23].

Recent investigations have been focused on the mechanisms of action of ART-based cancer therapy. We and others have reported that the ART compounds exert their anticancer effects by inhibiting cell proliferation, inducing cell cycle arrest and apoptosis, inhibiting angiogenesis, reducing cell migration and invasion [14–16, 24, 25]. However, the molecular targets and underlying mechanisms of action for ART derivatives as anticancer agents remain to be elucidated. In the present study, we tested the hypothesis that platelet-derived growth factor receptor-alpha (PDGFRα) is a key molecular target for DHA as a selective anti-PDGFRα-positive ovarian cancer agent.

## Results

### An overview of the results of a quantitative proteomics analysis of DHA-related changes in protein expression

We have previously reported that DHA inhibits the growth and induces apoptosis in human A2780 and OVCAR3 ovarian cancer cells [15]. To facilitate the identification of the potential cellular targets of DHA, we performed a proteomic analysis of the DHA-related changes in protein expression in A2780 cells treated with 10 μM of DHA for 24 h by using iTRAQ-labeled LC-MS. A total of 9659 unique spectra were matched to 2157 proteomes after data filtering to eliminate the low scoring spectra. Using a cutoff of a 1.5-fold change for two unique peptides, we identified 130 unique proteins that were differentially expressed between the control and DHA-treated cells. Of the 130 unique proteins found to have been affected by DHA treatment, we identified 21 membrane receptor proteins (Supplementary Table S1), 13 proteins related to cell migration and the epithelial–mesenchymal transition (EMT) (Supplementary Table S2), 67 proteins related to apoptosis and cell cycle regulation, 17 cytokines and chemokines, and 12 enzymes involved in biological synthesis and metabolism (data not shown). As shown in Supplementary Table S1, DHA markedly decreased the protein level of multiple receptor tyrosine kinases (such as PDGFRα/β, EGFR and VEGFR1/2/3), integrins (integrin β1/β2), toll-like receptors (TLR2/4/9) and other membrane proteins.

### PDGFRα is a potential molecular target of DHA

**Figure 1:**
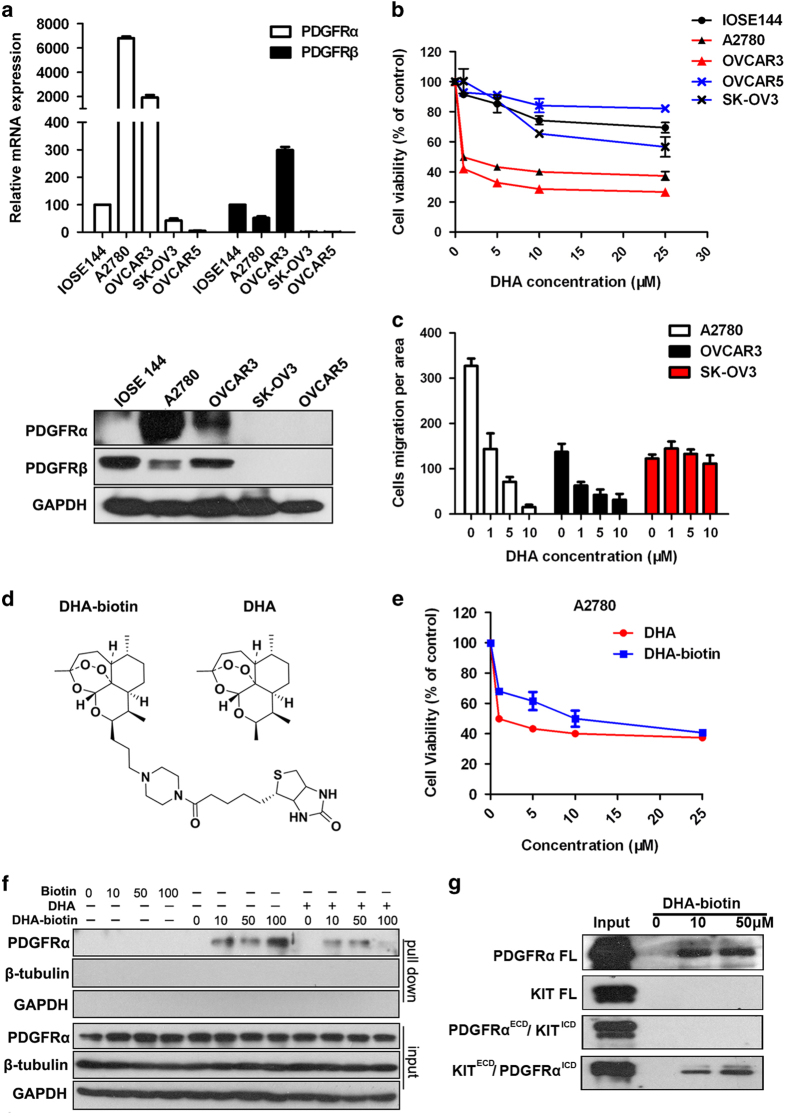
DHA targets PDGFRα and selectively inhibits the growth and migration of PDGFRα-positive ovarian cancer cells. (**a**) The mRNA and protein expression of PDGFRα and PDGFRβ in human A2780, OVCAR3, SK-OV3 and OVCAR5 ovarian cancer cells, and the non-malignant ovarian epithelial cell line, IOSE144. Glyceraldehyde-3-phosphate dehydrogenase (GAPDH) was used as an internal control. (**b**) The results of the CCK-8 assay evaluating the viability of ovarian cancer cells and non-malignant cells after a 48-h exposure to DHA. **(c**) The results of the cell migration assay using A2780, OVCAR3 and SK-OV3 cells treated with DHA for 12 h. (**d**) The chemical structures of DHA and biotinylated DHA (DHA-biotin). (**e**) The viability of A2780 cells after incubation with various concentrations of DHA and biotinylated DHA (DHA-biotin) for 48 h. (**f**) The direct interaction of DHA with solubilized endogenous PDGFRα. A2780 cell lysates were incubated with free biotin or biotin-labeled DHA at various concentrations (0, 10, 50, 100 μM), which were then conjugated with neutroavidin-agarose beads in the presence or the absence of 50 μM of non-labeled DHA. The bound protein was detected using an anti-PDGFRα antibody. The data are representative of more than three experiments with similar results. (**g**) DHA-biotin directly binds the PDGFRα intercellular domain. The 293T cells transfected with full-length (FL) PDGFRα or c-KIT, or the chimera receptors, and the cell lysates were incubated with biotin-labeled DHA at indicated concentration followed by streptavidin pulldown and immunoblotting analysis. The data shown are the means±s.e.m. for three independent experiments. PDGFR, platelet-derived growth factor receptor; DHA, dihydroartemisinin.

Membrane proteins are the hallmark of a cancer cell and significant targets for drug discovery, due to their unique and important role in cellular communications and signal transduction [26]. On the basis of our proteomic analysis, we suspected that DHA might target some receptor protein(s), and inhibit the downstream signaling of that receptor, thus decreasing the cancer cell growth and metastasis. We first analyzed the expression of DHA-regulated receptors in human A2780, OVCAR3, SK-OV3 and OVCAR5 ovarian cancer cells and the non-malignant ovarian epithelial cell line, IOSE144 (Figure 1a and Supplementary Figure S1), and examined whether there was a relationship between the inhibitory effects of DHA on cell growth and migration and the expression of these receptors. Interestingly, the level of PDGFRα, an important receptor tyrosine kinase involved in the development and progression of ovarian cancer [2, 27–30], was correlated with the sensitivity to DHA-induced cytotoxicity, while there was no correlation for other receptors including PDGFRβ, VEGFRs, EGFR,TLRs, ERα, AR, integrins, PGRMC1 (Figure 1a and Supplementary Figure S1). DHA could selectively inhibit the growth (Figure 1b) and migration (Figure 1c) of PDGFRα-positive ovarian cancer cells (A2780 and OVCAR3), with significantly decreased effects on PDGFRα-null SK-OV3, OVCAR5 and IOSE144 cells (Figure 1b and c). We further used an affinity protein purification approach [31, 32] to verify whether DHA could directly bind to PDGFRα. To purify the potential target protein, we synthesized a biotinylated derivative of DHA (DHA-biotin) (Figure 1d) that had similar inhibitory effects on ovarian cancer cells as were seen with the unlabeled DHA (Figure 1e). A2780 cell extracts were incubated with different concentrations of DHA-biotin or free biotin conjugated with streptavidin-conjugated agarose beads. The bound proteins were separated by SDS–PAGE after extensive washing, and were detected by western blotting; the results showed that DHA-biotin selectively bound to PDGFRα in a concentration-dependent manner (Figure 1f). Moreover, the binding of DHA-biotin to PDGFRα could effectively compete with that of the unlabeled DHA (Figure 1f).

As members of Class III receptor tyrosine kinases, c-KIT and PDGFRα share sequence homology and a similar overall structure [33]. To facilitate the identification of the binding domain to DHA, we generated two chimeric receptors containing the PDGFRα extracellular domain (ECD) fused to the transmembrane and cytoplasmic domain (ICD) of the c-KIT (named as PDGFRα^ECD^/KIT^ICD^) or c-KIT ECD fused to the PDGFRα intercellular domain (named as KIT^ECD^/PPDGFRα^ICD^), respectively. We showed that DHA-biotin directly binds to full-length PDGFRα and the KIT^ECD^/PPDGFRα^ICD^ chimeric receptor, respectively (Figure 1g), but not to the full-length c-KIT or PDGFRα^ECD^/KIT^ICD^ (Figure 1g).

Taken together, these results demonstrated that PDGFRα is the cellular target of DHA.

### DHA decreases PDGFRα expression by inducing its ubiquitination and proteasomal degradation

**Figure 2:**
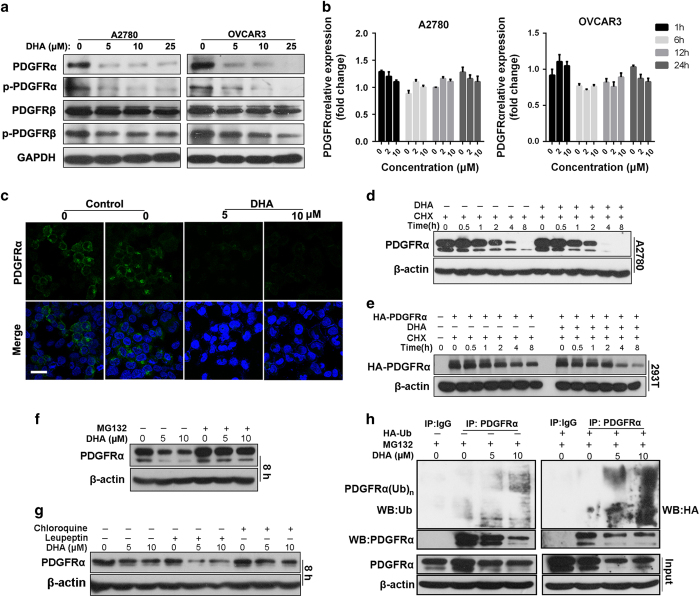
DHA induces PDGFRα ubiquitination and proteasomal degradation. (**a**) A2780 and OVCAR3 cells were exposed to various concentrations of DHA for 24 h, followed by a western blotting assay. (**b**) The *PDGFRA* mRNA expression level of the cells at various time points after exposure to DHA. (**c**) Immunostaining of PDGFRα on the A2780 cell membrane after exposure to DHA for 24 h. The green signals represent PDGFRα staining, and the blue signals indicate the cell nuclei. Scale bar, 20 μm. (**d**, **e**) A2780 cells (**d**) or 293T cells (PDGFRα null) transiently transduced with the control vector or HA-tagged PDGFRα vector (**e**) were treated with 50 mg ml^−1^ of cycloheximide (CHX) followed by exposure to 10 μM of DHA or dimethyl sulfoxide (DMSO). (**f**, **g**) 10 μM of MG132 (**f**) or 50 μM of chloroquine or leupeptin (**g**) was added to the DHA-treated A2780 cells 6 h before collecting the cell lysates. (**h**) A2780 cells were transiently transfected with the control vector or HA-tagged ubiquitin-expressing vector for 36 h, and then were treated with various concentrations of DHA for another 24 h. MG132 was added 6 h before the immunoprecipitation assay was performed to induce the accumulation of the ubiquitinated PDGFRα. DHA, dihydroartemisinin; PDGFR, platelet-derived growth factor receptor.

DHA treatment led to a marked decrease in the protein levels of total PDGFRα and its phosphorylated form, while the PDGFRβ levels were not significantly affected (Figure 2a). However, no significant effect of DHA treatment on the *PDGFRA* mRNA transcription level was observed (Figure 2b). The immunofluorescent staining also confirmed that there was significantly decreased expression and membrane location of the PDGFRα protein in A2780 cells after DHA exposure (Figure 2c).

To elucidate how DHA reduces the PDGFRα protein level, A2780 cells were pre-treated with a protein synthesis inhibitor, cycloheximide (CHX; 50 μg ml^−1^), followed by exposure to 10 μM of DHA or vehicle control, and the protein level of PDGFRα was analyzed at different time points. DHA increased the degradation rate of the endogenous PDGFRα protein compared to vehicle treatment (Figure 2d). Similarly, DHA also accelerated the degradation of exogenous PDGFRα protein in 293T cells (PDGFRα-null) which were transiently transfected with hemagglutinin (HA)-tagged PDGFRα expression vector (Figure 2e). We then investigated whether the decreased PDGFRα protein stability was induced by the ubiquitination and proteasomal degradation of the receptor. We found that the decrease in PDGFRα expression induced by an 8-h incubation with DHA was significantly inhibited by treatment with MG132, a proteasome inhibitor (Figure 2f) but not by lysosomal proteases inhibitors chloroquine or leupeptin (Figure 2g). Consistently, a subsequent ubiquitination assay revealed that the endogenous PDGFRα ubiquitination was increased after DHA treatment (Figure 2h).

### The DHA-induced suppression of cell growth and repression of the EMT are dependent on the downregulation of PDGFRα

**Figure 3:**
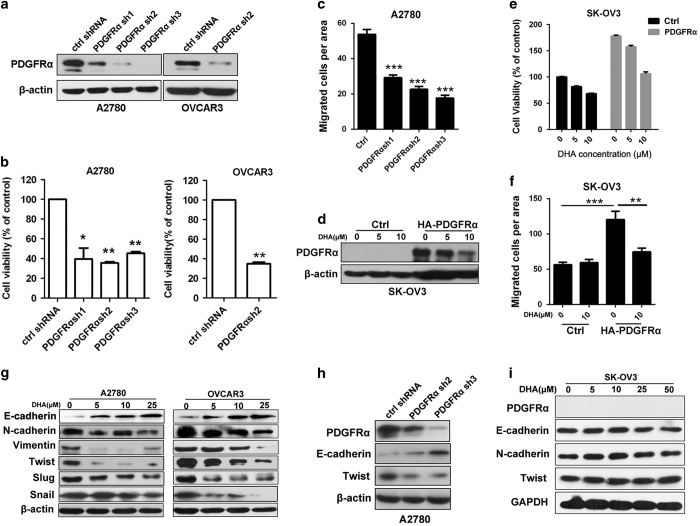
PDGFRα mediates the DHA-induced suppression of cancer cell growth, the EMT and migration. (**a**) A2780 and OVCAR3 cells treated with control or different lentivirus-mediated PDGFRα shRNAs for 72 h. (**b**) Cell viability was detected in A2780 and OVCAR3 cells after knocking down of PDGFRα. (**c**) Cell migration was evaluated in A2780 cells with or without knockdown of PDGFRα. (**d**) SK-OV3 cells stably expressing PDGFRα were treated with DHA for 24 h. The expression of PDGFRα was detected by western blotting. (**e**, **f**) Cell viability (**e**) and migration (**f**) was detected in SK-OV3 cells overexpression of PDGFRα after incubation with DHA. (**g**) The expression of the EMT-related proteins were detected in A2780 or OVCAR3 cells after incubation with DHA for 24 h. (**h**) The expression of E-cadherin and twist in A2780 cell with knockdown of PDGFRα. (**i**) EMT-related proteins detected in the SK-OV3 (PDGFRα null) cells after a 24-h exposure to DHA. The data shown are representative of values from at least three independent experiments with similar results (means±s.e.m.; *P<0.05; **P<0.01; ***P<0.001). DHA, dihydroartemisinin; PDGFR, platelet-derived growth factor receptor; EMT, epithelial–mesenchymal transition.

To further demonstrate that PDGFRα inhibition is responsible for the inhibitory effects of DHA on cell growth and migration, we silenced the expression of PDGFRα in A2780 and OVCAR3 cells using specific shRNAs (Figure 3a), and found that PDGFRα knockdown led to cell growth arrest (Figure 3b) and repressed cell migration (Figure 3c). The exogenous PDGFRα stable expressing SK-OV3 cells were generated and then were tested for sensitivity to DHA. As shown in Figure 3d, DHA decreased the expression of exogenous PDGFRα in a dose-dependent manner. SK-OV3 cells expressing PDGFRα showed enhanced growth and migration ability related to the cell stably transfected with control vector (Figure 3e and f). Treatment with DHA could significantly decrease the growth and motility of PDGFRα-expressing SK-OV3 cells but had less effect on PDGFRα-null cells (Figure 3e and f).

Our proteomic data had shown that DHA could regulate several EMT-related proteins (Supplementary Table S2), which have been documented to augment the motility of ovarian cancer cells [34]. It has been reported that multiple membrane receptors, including PDGFRα, could provoke EMT progression and enhance tumor metastasis [35, 36]. Based on these observations, we examined whether the inhibitory effects of DHA on the migration of ovarian cancer cells was linked to the impairment of PDGFRα-mediated EMT signals. We found that A2780 and OVCAR3 cells treated with DHA exhibited a repressed EMT phenotype, including the upregulation of an epithelial marker, E-cadherin, and downregulation of mesenchymal markers, N-cadherin and vimentin, and the transcriptional repressors Snail, Twist, and Slug (Figure 3g and Supplementary Figure S2A and B). The protein level of PDGFRα was positively correlated with the EMT status (Supplementary Figure S2C), and PDGFRα knockdown functioned in a similar manner to DHA exposure, enhancing the expression of E-cadherin and downregulating the expression of its transcriptional repressor, Twist (Figure 3h and Supplementary Figure S2D). Of note, DHA had no effect on the expression of EMT-related proteins in PDGFRα-null SK-OV3 and OVCAR5 cells (Figure 3i and Supplementary Figure S2E), suggesting that DHA inhibited cell migration specifically by impairing the PDGFRα-mediated EMT signaling.

Taken together, all of these findings demonstrate that DHA targets PDGFRα to inhibit ovarian cancer cell growth, the EMT and cell migration.

### The inactivation of the phosphatidyl-inositol-3-kinases (PI3Ks)/protein kinase B (AKT) and mitogen-activated protein kinase (MAPK)/extracellular signal-regulated kinase (ERK) pathways is involved in the effects of DHA on ovarian cancer cells

**Figure 4:**
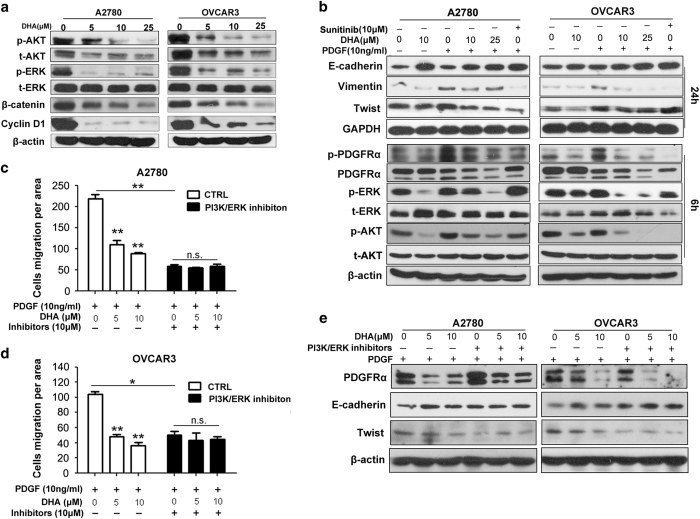
DHA inhibits the PI3K/AKT and MAPK-ERK pathways by suppressing PDGFRα. (**a**) The results of an immunoblotting assay showing the phosphorylation state of PI3K/AKT and MAPK-ERK signaling molecules and their downstream proteins, β-catenin and cyclin D1 expression, in A2780 and OVCAR3 cells treated with different concentrations of DHA for 24 h. (**b**) The cells were pretreated with or without 10 ng ml^−1^ of PDGF-BB for 0.5 h, followed by incubation with DHA or control for 6 or 24 h. Sunitinib was used as a positive control from PDGFRα inhibition. The expression of EMT-related proteins and the activation of PDGFRα, AKT and ERK were examined by western blotting. (**c**, **d**) Transwell assays were performed using cells pretreated with 10 ng ml^−1^ of PDGF-BB, following a 12-h exposure to DHA in the presence or absence of the PI3K inhibitor LY29002 (10 μM) and MEK1 inhibitor PD98059 (10 μM) (means±s.e.m.; *P<0.05, **P<0.01; *n*=3). (**e**) The expression levels of PDGFRα and EMT phenotype markers detected by western blotting. Cells pre-stimulated with 10 ng ml^−1^ of PDGF-BB were exposed to the indicated concentrations of DHA for 24 h, followed by the addition of 10 μM of LY294002 and PD98059. DHA, dihydroartemisinin; PI3K, phosphoinositide 3-kinase; MAPK, mitogen-activated protein kinase; PDGFR, platelet-derived growth factor receptor; EMT, epithelial–mesenchymal transition.

Considering that PI3K/AKT and MAPK pathways are the most important downstream pathways for PDGFRα signaling, and the fact that their activation is emerging as a central feature of the EMT phenotype [37], we investigated the involvement of these two pathways in the DHA-induced growth and metastasis suppression. PI3K/AKT and MAPK pathways were significantly inactivated by DHA, in a dose- and time-dependent manner, as evidenced by the dephosphorylation of AKT and ERK, and the subsequent repression of downstream target proteins, including β-catenin and cyclin D1 (Figure 4a and Supplementary Figure S3A and B). The AKT expression was also decreased (Figure 4a). Further studies revealed that DHA repressed the PDGF-stimulated phosphorylation of PDGFRα and the activation of AKT and ERK (Figure 4b), and subsequently reversed the PDGF-induced EMT phenotype (Figure 4b). These effects were similar to those seen following treatment with Sunitinib, the PDGFRα inhibitor used as a positive control in our studies (Figure 4b). Interestingly, we found that DHA had no effect on the EGF-induced phosphorylation of AKT and ERK in PDGFRα-null SK-OV3 cells (Supplementary Figure S3C).

To determine whether constitutive activation of PI3K/AKT and MAPK pathway prevents the inhibitory effect of DHA in PDGFRα-positive ovarian cancer, A2780 cells were transiently transfected with constitutively active AKT (Myc-tagged CA-AKT) expressing vector or/and constitutively active K-RAS (GFP-tagged K-RAS G12V) vector (Supplementary Figure S4A). As shown in Supplementary Figure S4B, constitutive PI3K/AKT signaling and RAS-MAPK signaling attenuated the cell growth inhibition led by DHA. In addition, we further investigated whether the inactivation of the PI3K/AKT and MAPK pathways was involved in the inhibitory effects of DHA on cell migration and the EMT. Suppression of both pathways led to decreased mobility in both the A2780 and OVCAR3 cells, and the effects were similar to those of DHA exposure (Figure 4c and d). Moreover, the inactivation of the PI3K/AKT and MAPK pathways attenuated the effects of DHA on cell migration (Figure 4c and d). The western blotting results also revealed that, although the inactivation of the PI3K/AKT and MAPK pathways had no major impact on the decrease in the expression of PDGFRα caused by DHA treatment, it did attenuate the effects of DHA on the expression of EMT-related proteins (Figure 4e).

Together, these findings suggest that the inactivation of the PI3K/AKT and MAPK pathways is involved in the inhibitory effects of DHA on cell growth and migration, and that this occurs via the inhibition of PDGFRα activity and stability.

### DHA inhibits ovarian cancer cell growth and metastasis, and sensitizes ovarian cancer cells to PDGFR inhibitors *in vivo*

**Figure 5:**
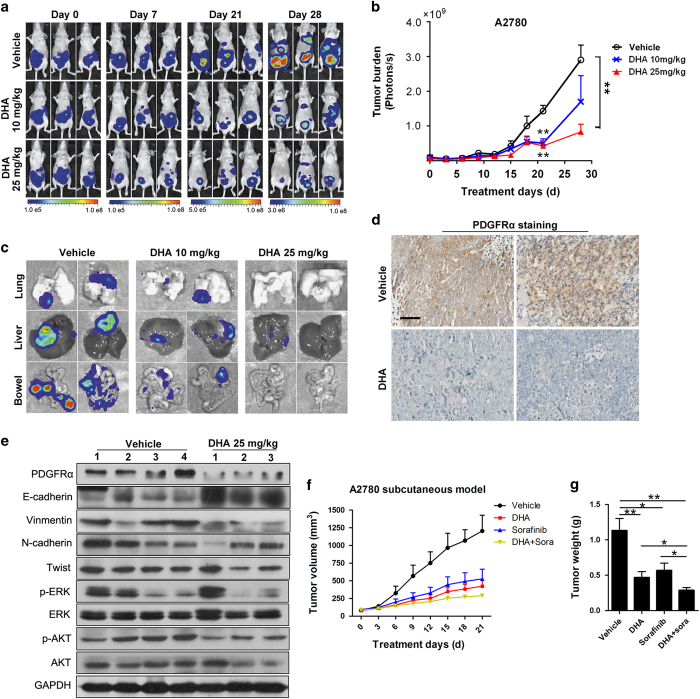
DHA inhibits ovarian cancer cell growth and metastasis, and sensitizes ovarian cancer cells to PDGFR inhibitor *in vivo*. (**a**, **b**) Representative images (**a**) and quantification bioluminescence (**b**) of A2780-luci bearing mice treated with DHA (10 mg kg^−1^ or 25 mg kg^−1^) or the vehicle (means±s.e.m.; *P<0.05. **P<0.01; *n*=8). (**c**) Representative bioluminescence images of different organs with metastatic or disseminated cancer cells. (**d**) Immunohistochemical staining of PDGFRα in the tumors after DHA treatment. (**e**) Western blot analysis showing the expression of PDGFRα, pAKT, pERK and EMT-related protein in tumors treated with DHA (25 mg kg^−1^) or the vehicle. The Arabic numbers indicate individual tumors. **(f**) The tumor growth in subcutaneous A2780 tumor bearing mice treated with DHA 30 mg kg day^−1^ (i.p.), Sorafinib 30 mg kg day^−1^ (intragastric administeration, i.g.), or the combination of the two. (**g**) The subcutaneous tumors were harvested and weighed. Data are shown as means±s.e.m. (*n*=5; *P<0.05, **P<0.05). DHA, dihydroartemisinin; PDGFR, platelet-derived growth factor receptor; EMT, epithelial–mesenchymal transition.

We further investigated the *in vivo* effects of DHA on ovarian cancer growth and metastasis by using nude mice that developed tumor in organs throughout the peritoneal space after i.p. injection of luciferase-labeled A2780 cells. The mice in the control group developed tumors in organs throughout the peritoneal space and pectoral cavity, and the bioluminescence signal progressively increased with time, while the tumor progression was significantly inhibited in the mice that received 10 or 25 mg kg^−1^ of DHA (Figure 5a and b). DHA-treated mice developed fewer metastases and/or had fewer disseminated cancer cells in various organs (lungs, liver and bowel) than the control animals (Figure 5c and Supplementary Figure S5A). Immunohistochemical results showed a marked downregulation of PDGFRα expression in the tumor samples from animals treated with DHA (Figure 5d). Western blotting with tumor tissue samples also showed that treatment with DHA led to marked decreases in the expression of PDGFRα, and inactivation of AKT and ERK and the repression of the EMT phenotype, that is, the upregulation of E-cadherin and downregulation of N-cadherin and vimentin and twist (Figure 5e).

Many PDGFR inhibitors including sorafenib, sunitinib, and neutralizing PDGFR antibodies are being investigated in clinical trials in patients with recurrent ovarian cancer [30]. Based on our aforementioned findings, we have hypothesized that DHA may enhance tumor cell sensitivity to the clinical PDGFR inhibitors. To address this possibility, the efficacy of DHA in combination with sorafinib or sunitinib was examined in A2780 xenograft tumor model. We noted that single-agent DHA or sorafinib slowed tumor growth (Figure 5f and g). Interestingly, a significantly improved effect from combination therapy compared to either single-agent DHA or single-agent Sorafinib was observed (Figure 5f and g). Of note, combined treatment led to more protein degradation of PDGFRα and better inactivation of PI3K/AKT and MAPK signaling (data not shown). Similarly, mice treated with the combination agents DHA and Sunitinib also exhibited an improved tumor growth inhibition (Supplementary Figure S5B and C). These results indicated the feasibility of DHA as a sensitizer for PDGFR-targeted therapy for ovarian cancer patients.

### PDGFRα expression in human ovarian cancer tissues is a prognosticator of patients overall survival and is associated with advanced disease and metastasis

**Figure 6:**
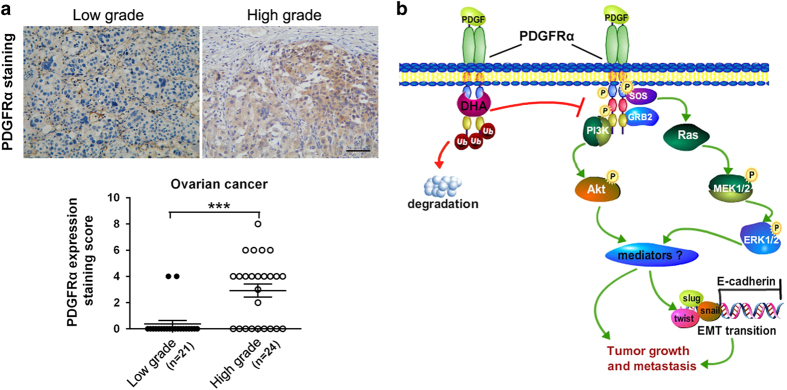
The PDGFRα expression is elevated in human metastatic ovarian cancer tissues. (**a**) The immunostaining intensity score of the PDGFRα expression in ovarian tumor cells. Forty-five surgical specimens from ovarian cancer patients (21 low grade cases with no obvious metastasis, and 24 high grade cases with extensive metastasis) were immunostained with a PDGFRα antibody. Scale bar, 100 μm (means±s.e.m.; ***P<0.001). (**b**) A schematic representation of the major molecular mechanism of action for the effects of DHA on ovarian cancer growth and metastasis. PDGFR, platelet-derived growth factor receptor; DHA, dihydroartemisinin.

To further demonstrate the role of PDGFRα in ovarian cancer development and progression, and to confirm its clinical relevance, we collected four independent cohorts of ovarian cancer from Gene Expression Omnibus (GEO) and The Cancer Genome Atlas (TCGA) data sets for which genome wide gene expression and survival data were publicly available, and examined how the PDGFRα expression correlates with patient survival. The results showed that, for four cohorts, there was an inverse association between PDGFRα expression and patients overall survival (Supplementary Figure S6). We further examined the correlation of PDGFRα expression levels to the metastasis status of ovarian cancer patients. The paraffin-embedded epithelial ovarian tumor specimens (*n*=45) were selected according to their pathologic diagnoses, including 21 low grade (I–II) cases with no obvious lymphatic and abdominal metastasis, and 24 high grade (III–IV) cases with extensive lymphatic and abdominal metastasis (Supplementary Table S3). The quantitative analysis of the IHC staining results revealed that the PDGFRα expression was significantly enhanced in the tumor cells ovarian cancer patients with high metastasis (Figure 6a and Supplementary figure S7; Supplementary Table S3). These results indicated that PDGFRα may be a prognostic biomarker and a potential therapeutic target for ovarian cancer.

## Discussion

In the present study, we identified and validated PDGFRα as a novel molecular target for DHA, which was based on the following major findings. First, DHA selectively inhibits the growth and migration of PDGFRα-positive ovarian cancer cells. Second, DHA inhibited PDGFRα expression via promoting its protein degradation. Third, following PDGFRα downregulation by DHA, the downstream PI3K/AKT and MAPK/ERK signaling pathways were inactivated and the EMT was repressed, resulting in suppression of the tumor growth and migration. Fourth, the effects of DHA on the metastasis of ovarian cancer cells were confirmed *in vivo*. Fifth, DHA sensitizes ovarian cancer cells to clinically used PDGFR inhibitors *in vivo*. Our initial clinical molecular pathology studies further demonstrated that there is linkage between high PDGFRα expression and advanced disease and metastasis in ovarian cancer patients. Figure 6b depicts our proposed model for the effects of DHA on ovarian cancer cells and its mechanisms of action, based on the aforementioned *in vitro* and *in vivo* findings.

PDGFR (α and β) is overexpressed in a high percentage of human malignancies, including ovarian cancer; its activation promotes cell growth, proliferation and metastasis [29, 38–43]. Generally, when PDGFR is activated by PDGF stimulation, it can interact with and activate proteins with SH2 domains, subsequently activating the PI3K, Ras-MAPK and phospholipase γC (PLC) pathways and contributing to tumor growth and progression [42]. Accumulating evidence from both animal experiments and human clinical studies suggests that PDGFRα is involved in the progression of ovarian cancer [27–29, 39, 43]. Our results demonstrated that DHA, one of the most effective ART derivatives, promoted the degradation of PDGFRα protein and selectively inhibited the growth and migration and EMT of PDGFRα-positive ovarian cancer cells. We provided further evidence supporting the role of PDGFRα in cell growth and motility by performing experiments with knockdown and exogenous overexpression of PDGFRα protein. We also found that there was increased expression of PDGFRα in ovarian cancer patients with high pathobiological grade and metastatic disease compared with their less aggressive counterparts. Recent reports also indicate that the PDGF-PDGFR system is involved in the EMT process [37, 44].

Recent studies uncovered the role of protein ubiquitylation in vesicular trafficking of growth factor receptors [45, 46]. Previous studies that analyzed downregulation of PDGFRα focused the attention on Cbl-family ubiquitin ligases as a modifier of receptor ubiquitylation [47]. There are considerable efforts being made to validate PDGFR as a novel cancer target, and several inhibitors of PDGFR and related kinases are being developed or have already entered clinical trials for women with ovarian cancer, such as imatinib, sorafinib and sunitinib [38–41,  48]. Compared with these PDGFRα kinase inhibitors, DHA distinguished itself by exerting its anticancer activity mainly via accelerating PDGFRα protein degradation. One possibility is that DHA binds to PDGFRa and changes its spatial conformations and structure. These modifications may increase the affinity of PDGFRa to its E3 enzyme and, accordingly, enable efficient ubiquitination of this receptor. We also demonstrated a significantly improved effect from combination therapy with DHA and sorafinib or sunitinib, indicating the feasibility of DHA as a sensitizer for PDGFR-targeted therapy for ovarian cancer patients.

The results from the present study may have high potential for translation to clinical practice. Our results uncovered a previously unknown target of DHA responsible for its effects on cell growth and migration in ovarian cancer cells. We speculate that the inhibition of PDGFRα by DHA is important for its actions on not only cell growth, proliferation and migration, but also apoptosis and angiogenesis [14, 49], which would facilitate the rational design of effective DHA-based cancer therapy. Considering that ART compounds are currently clinically used drugs with favorable safety profiles, the results from this study will potentate their use in combination with clinically used PDGFRα inhibitors, leading to maximal therapeutic efficacy with minimal adverse effects in PDGFRα-positive cancer patients. We also believe that the results from the current study will facilitate the discovery of novel DHA-based, PDGFRα-targeting agents for the treatment of ovarian cancer, and possibly for other cancers as well.

## Materials and methods

### Cell culture and reagents

Ovarian cancer cell lines OVCAR3, A2780 and SK-OV3 were obtained from ATCC, which were authenticated by short-tandem repeat analysis by the ATCC. Ovarian cancer cell line OVCAR5 was generously provided by Dr Geng Meiyu (Chinese Academy of Sciences). The IOSE144, OVCAR3, A2780 and OVCAR5 cells were cultured in RPMI 1640 medium, the SK-OV3 cells were cultured in Dulbecco's modified Eagle medium (DMEM) medium, with all media containing 10% fetal bovine serum, 100 units ml^−1^ of penicillin, and 100 mg ml^−1^ of streptomycin. The cells were incubated at 37 °C with 5% CO_2_.

DHA, sunitinib, chloroquine, leupeptin and MG132 were purchased from Sigma (St Louis. MO, USA). Sorafenib was purchased from Santa Cruz Biotechnology (Santa Cruz, CA, USA). The kinase inhibitors LY294002 and PD98059 were obtained from Cell Signaling (Beverly, MA, USA).

### Quantitative iTRAQ LC-MS/MS proteomic analysis

A2780 cells were treated with the vehicle control or 10 μM DHA for 24 h. The iTRAQ labeling of the peptide samples derived from the control and DHA-treated cells was performed and separated by nanoflow LC and analyzed by Q Exactive MS (Thermo Finnigan, San Jose, CA, USA) (further details in Supplementary Methods).

### Cell viability assay

The cell viability was determined by using the CCK-8 assay. Briefly, 4000 cells were plated in triplicate in 96-well plates. After an overnight incubation, the cells were exposed to various concentrations of DHA or other ART analogs or to the control for the indicated times. After treatment, the cell viability was assessed by examining the formation of formazan crystals by evaluating the absorbance at 450 nm. The experiment was repeated at least three times under the same conditions.

### Cell migration and invasion assay

For the cell migration assay, cells were seeded onto Transwell Permeable Support inserts with an 8 μm microporous membrane (Corning Costar: Tewksbury MA, USA) in 24-well plates. Culture medium with 10% fetal bovine serum was used as a chemoattractant in the lower compartment. Within 8–12 h, non-invasive cells were carefully removed, and the cells that had migrated to the lower surface were fixed and stained with crystal violet or eosin. Then, the stained cells were counted (in 8 random 100× fields per well). For the invasion assay, the transwell was pre-coated with Matrigel (Becton Dickinson Labware, Franklin Lakes, NJ, USA). Each experiment was repeated at least three times.

### The generation of the human ovarian cancer localized and disseminated xenografts

For the localized model, 1×10^6^ A2780 cells were injected subcutaneously into the right flanks of 4-week old female Balb/c nude mice (obtained from Shanghai Slac Laboratory Animal Co., Shanghai, China). Mice bearing tumors about 0.5 cm in diameter were randomized into control and treatment groups (*n*=5). Tumor mass was determined by caliper measurements using the formula ‘1/2*a*×*b*^2^’, where ‘*a*’ is the long diameter and ‘*b*’ is the short diameter (in cm). For dissemination, 4-week-old female BALB/c nude mice were injected i.p. with 3×10^6^ A2780 cells labeled with luciferase, in which the gross pathology and pattern of dissemination of tumor nodules resemble human metastatic ovarian cancer . Optical imaging to determine the luciferase expression in the mouse model was conducted using the IVIS Lumina Bioluminescence Imaging System. Mice bearing tumors were equally grouped into the treatment and control groups (8/group) according to the level of bioluminescence. DHA, dissolved in Cremophor EL:Ethanol:saline (5:5:90, V/V/V), was i.p. injected at doses of 10 mg kg^−1^ or 25 mg kg^−1^, 5 days per week, for 4 weeks. The animal use and experimental protocols were reviewed and approved by the Institutional Animal Care and Use Committee (IACUC) of the Institute for Nutritional Sciences.

### Statistical analysis

The migration and invasion assay and CCK-8 cell viability assay were performed for at least three independent experiments, and the results were presented as the means±s.e.m. Statistical significance was determined through a two-tailed Student’s *t*-test, with a *P*-value <0.05 considered to be statistically significant.
