# Supplementary material for: Dihydroartemisinin selectively inhibits PDGFRα-positive ovarian cancer growth and metastasis through inducing degradation of PDGFRα protein
Source: Cell Discov. 2017 Nov 21;3:17042–. doi: 10.1038/celldisc.2017.42 (PMC5787695; doi:10.1038/celldisc.2017.42)
Supplement: Supplementary Information [file celldisc201742-s1.pdf]

Supplementary materials:

Dihydroartemisinin selectively inhibits PDGFRα-positive ovarian cancer growth and metastasis through inducing degradation of PDGFRα protein

Table of contents

Supplementary Materials and Methods

Figure S1. The mRNA expression of membrane receptor proteins in ovarian cancer and non-malignant ovarian epithelial cells treated with DHA.

Figure S2. PDGFRα mediates the DHA-induced suppression of cancer cell growth, the EMT, and migration.

Figure S3. DHA selectively inactivates the PI3K/AKT and MAPK/ERK pathways in PDGFRα-positive cancer cells.

Figure S4. Constitutive activation of PI3K/AKT and MAPK/ERK attenuated the tumor killing effect of DHA in A2780 cells.

Figure S5. DHA inhibits ovarian cancer cell growth and sensitizes it to PDGFR inhibitor sunitinib in vivo.

Figure S6. For four cohorts, there is an inverse association between PDGFRα expression and patients overall survival.

Figure S7 The PDGFRα expression is elevated in human metastatic ovarian cancer tissues.

Table S1. The membrane receptor proteins differentially expressed after DHA exposure.

Table S2.The metastasis- and EMT-related proteins that were differentially expressed after DHA exposure.

Table S3. The clinicopathological characteristics of the patients and the expression of PDGFRα in ovarian cancer.

Table S4. The primary antibodies used in this study.

Table S5. The primers used in this study.

Supplementary Materials and Methods

Quantitative iTRAQ LC-MS/MS proteomic analysis. A2780 cells were treated with the vehicle control or 10 μM DHA for 24 hours (two biological replicates), and the protein lysates were digested with trypsin overnight. The iTRAQ labeling of the peptide samples derived from the control and DHA-treated cells was performed using an iTRAQ Reagent 8-plex kit (Applied Biosystems, USA). The labeled peptides were separated by nanoflow LC and analyzed by Q Exactive MS (Thermo Finnigan, USA). Briefly, peptides were separated using a nanobored C18 column (75μm x 100 mm, 3μm particles) at a flow rate of 0.25 μL/min. The mass spectrometer data were determined in the positive ion mode with a selected mass range of 300-1800m/z and a resolution of 70,000 at m/z 200. The MS raw data were analyzed by the Mascot2.2 and Proteome Discoverer1.3 software programs (Thermo Scientific, USA). Mascot2.2 was used to search the peak lists against the Human UniProtKB database (UniProtKB release 3 March 2013). Proteome Discoverer1.3 was used to quantity the proteins. Proteins with 1.5-fold or larger differences between DHA-treated and control samples were considered to be deferentially expressed proteins.

shRNA interference. For lentivirus-mediated PDGFRα silencing, three sequences of human PDGFRα shRNA in a FG12/GFP-shRNA lentivirus core vector were used as follows: sh1 TATAATGGCAGAATCATCAT; sh2 GCCTTTGTACCTCTAGGAATG; sh3 GGACATGAAGCAGGCTGATAC. Cells positively infected with PDGFR shRNA viral partials were sorted by FACS after incubation for 72 h under sterile conditions.

Western blotting

The immunoblot analysis was performed as described previously (1). Briefly, the

harvested cells were lysed for 30 min in ice-cold cell lysis buffer (Cell Signaling Technology, Inc) supplemented with 1 mM PMSF proteinase inhibitor. The lysate was centrifuged at 12,000 *g* for 15 min at 4 ℃. The supernatants of the extracts were measured by a protein assay using Bio-Rad DC reagents (Bio-Rad). Equal amounts of proteins were loaded into each lane of 10% SDS–PAGE gels, electrophoresed, and transferred to PVDF membranes (Millipore Corporation). The membranes were blocked in 1×TBST with 5% non-fat milk for 2 h, and were incubated overnight at 4 ℃ with primary antibody at a suitable dilution. After five washes with TBS/0.1% Tween-20 for a total of 60 min, the membrane was incubated with HRP-conjugated secondary antibody (Sigma) at a suitable dilution for 1.5 hours at room temperature. After five washes with TBS/0.1% Tween-20, detection was achieved using the Pierce ECL Western Blotting Substrate (Pierce) and Kodak X-Omat XAR film (Eastman Kodak Corporation) according to the manufacturer's instructions. The sources of the antibodies used in this study are listed in **Supplementary Table S4**.

RT-qPCR

Total cellular RNA (1μg) was extracted from the indicated cell lines using the Trizol reagent (Invitrogen), and was subsequently used as a template for reverse transcription (RT). Quantitative real-time PCR was performed on a Bio-Rad CFX96™ Real-Time PCR Detection System using SYBR Green as the detection fluorophore. The PCR amplification included a first step of 60 s at 95 ℃, followed by 40 cycles of amplification (95 ℃ for 15 s, 60 ℃ for 15 s and 72 ℃ for 30 s).The primer sequences are provided in **Supplementary Table S5**.

Immunohistochemical analysis

Forty-five tissue specimens of epithelial ovarian cancer were obtained from Nantong Tumor

Hospital, Jiangsu Province, China. The use of these human specimens was approved by the Ethics Committee of Nantong Tumor Hospital. Briefly, unstained 5.0 μm sections of clinical specimens were deparaffinized with xylene and rehydrated with ethanol, followed by staining with primary antibodies against PDGFRα (1:60), p-AKT (1:50), p-ERK (1:100) and E-cadherin (1:100). The immunohistochemical staining was analyzed using an Olympus microscope (Olympus) with the Q Capture Pro 6.0 software program. The immunohistochemical studies were conducted in a double-blind manner by a skilled pathologist, and the staining results were semi-quantitative by a third pathologist (Dr. Jianbing Zhang) by determining the immunoreactivity score (IRS) (2), which is a combination of the immunoreaction's intensity and the percentage of positive tumor cells. The staining pattern of PDGFRα was defined as low expression (IRS: 0-4) or high expression (IRS: 6-12) based on the IRS.

Supplementary references

1. Hou J, Wang D, Zhang R, Wang H. Experimental therapy of hepatoma with artemisinin and its derivatives: in vitro and in vivo activity, chemosensitization, and mechanisms of action. Clin Cancer Res 2008; 14: 5519-5530.

2. Remmele W, Stegner HE. Recommendation for uniform definition of an immunoreactive score (IRS) for immunohistochemical estrogen receptor detection (ER-ICA) in breast cancer tissue. Pathologe1987; 8:138-140.

Supplementary Figures

Figure S1

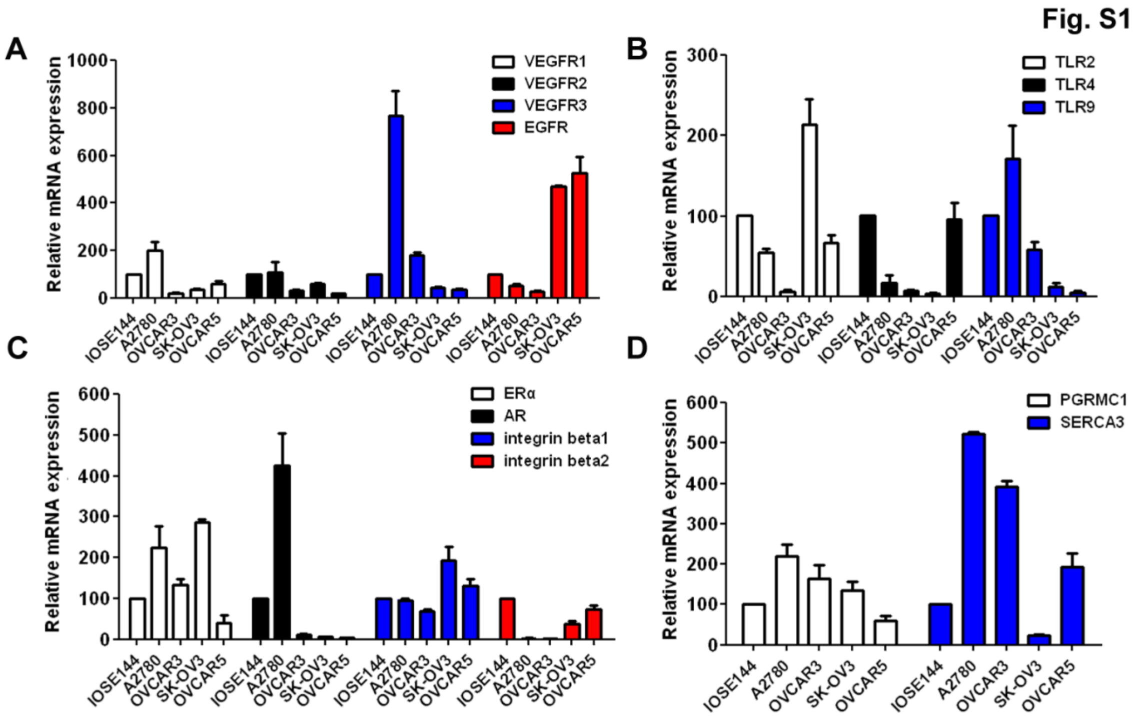

Figure S1 The mRNA expression of membrane receptor proteins in ovarian cancer and non-malignant ovarian epithelial cells.

The mRNA expression levels of the receptor tyrosine kinases **(A)**, toll-like receptors **(B)**, integrins and estrogen/androgen receptors **(C)** and other membrane proteins **(D)** were determined by real-time PCR. The results are the mean values ± SEM.

Figure S2

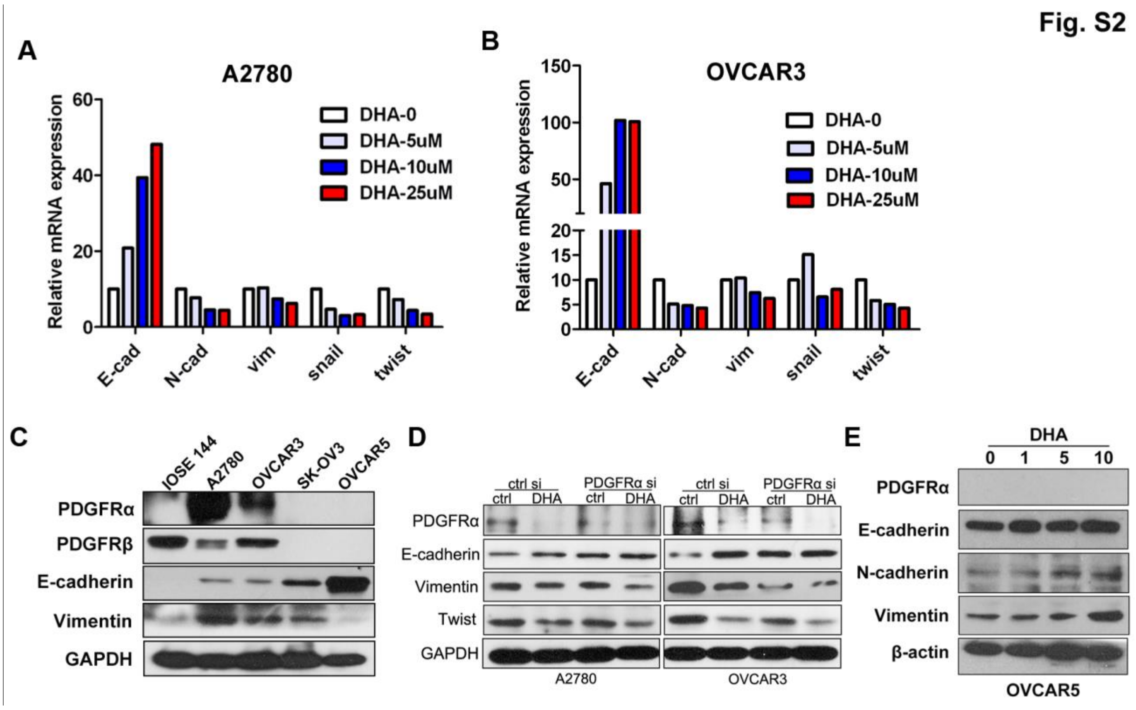

Figure S2 PDGFRα mediates the DHA-induced suppression of cancer cell growth, the EMT, and migration.

(A, B) The transcriptional changes in EMT-related genes in A2780 and OVCAR3 cells after a 24 h incubation with DHA, as detected by real-time PCR. **(C)** The expression of PDGFRα, PDGFRβ and EMT-related proteins in the indicated cell lines. (**D**) The expression of EMT related protein E-cadherin,vimentin and twist was detected in A2780 and OVCAR3 cells treated with control or PDGFRα siRNA, followed by exposure to 10 μM of DHA or control. **(E)** Western blotting analysis of EMT-related proteins in OVCAR5 (PDGFRa null) cells after treated with DHA at indicated concentration.

Figure S3

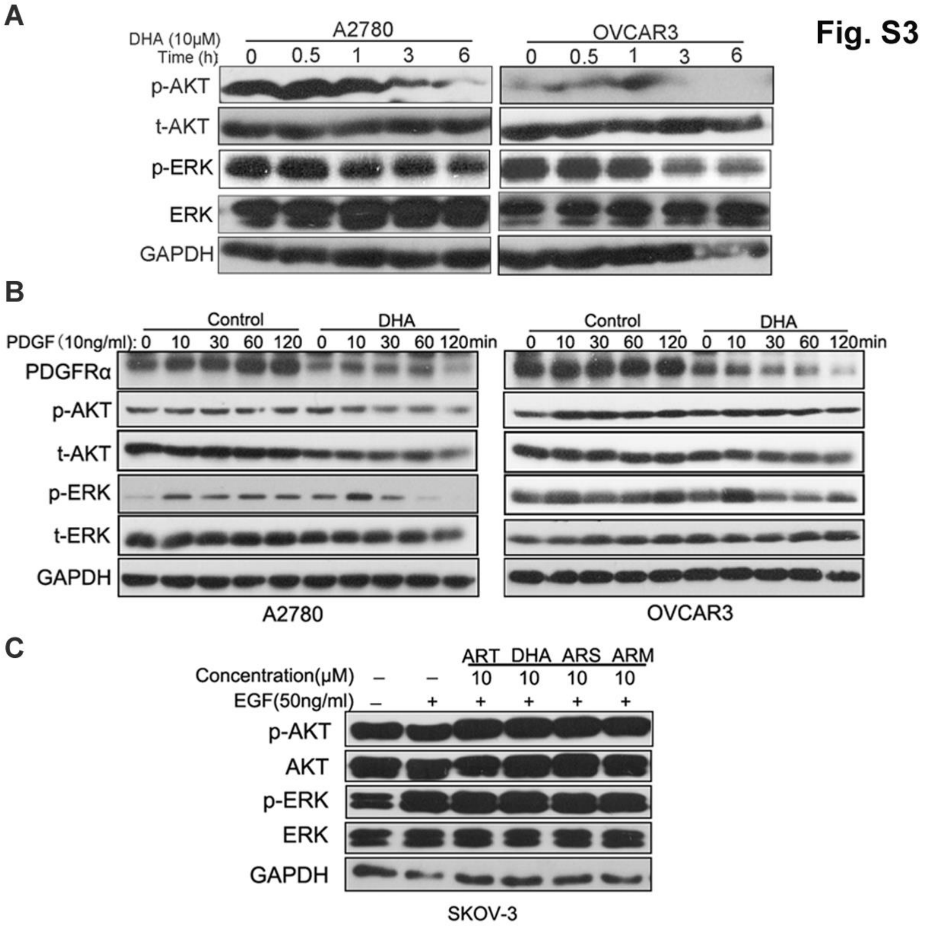

Figure S3 DHA selectively inactivates the PI3K/AKT and MAPK/ERK pathways in PDGFRα-positive cancer cells.

(A) The changes in the phosphorylation states of AKT and ERK in cells in a time-dependent manner after incubation with 10 μM of DHA. (**B)** A2780 and OVCAR3 cells pretreated with 10 μM of DHA for 20 h were stimulated with 10 ng/ml PDGF for the indicated times. The status of the PI3K/AKT and MAPK/ERK pathways was assessed by Western blot analyses. (**C)** The expression of AKT, ERK and their respectively phosphorylated forms in SK-OV3 cells after a 4-h exposure to 10 μM of DHA and other ART derivatives (ART, artemisinin; ARS, artesunate; and ARM, artemether) following a 50 ng/ml EGF pretreatment.

Figure S4

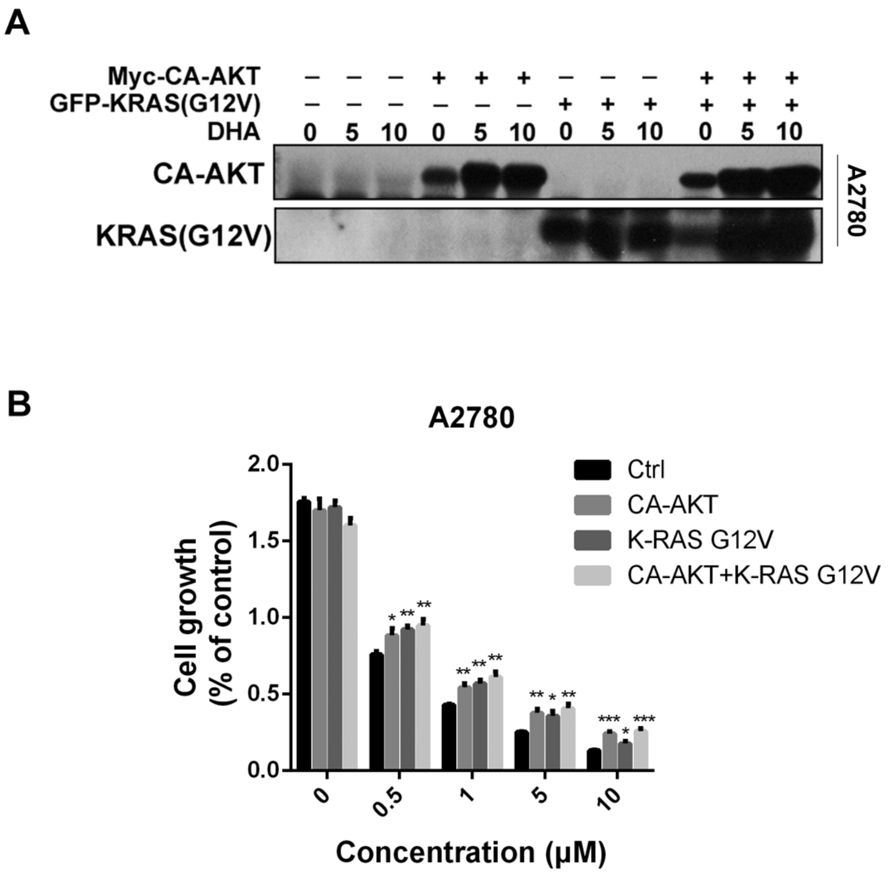

Figure S4 Constitutive activation of PI3K/AKT and MAPK/ERK pathway attenuated the tumor killing effect of DHA in A2780 cells. (A) A2780 cells were transiently transfected with control vector or vector encoding Myc-tagged constitutively activated AKT (CA-AKT), or vector encoding GFP-tagged constitutively activated K-RAS (K-RAS G12V), or both; Cells were then treated with DHA for 24 h. Western blot analysis showed the expression of proteins indicated. **(B)** Cell viability was detected in A2780 cells with or without constitutive activation of PI3K/AKT pathway or MAPK/ERK pathway or both.

Figure S5

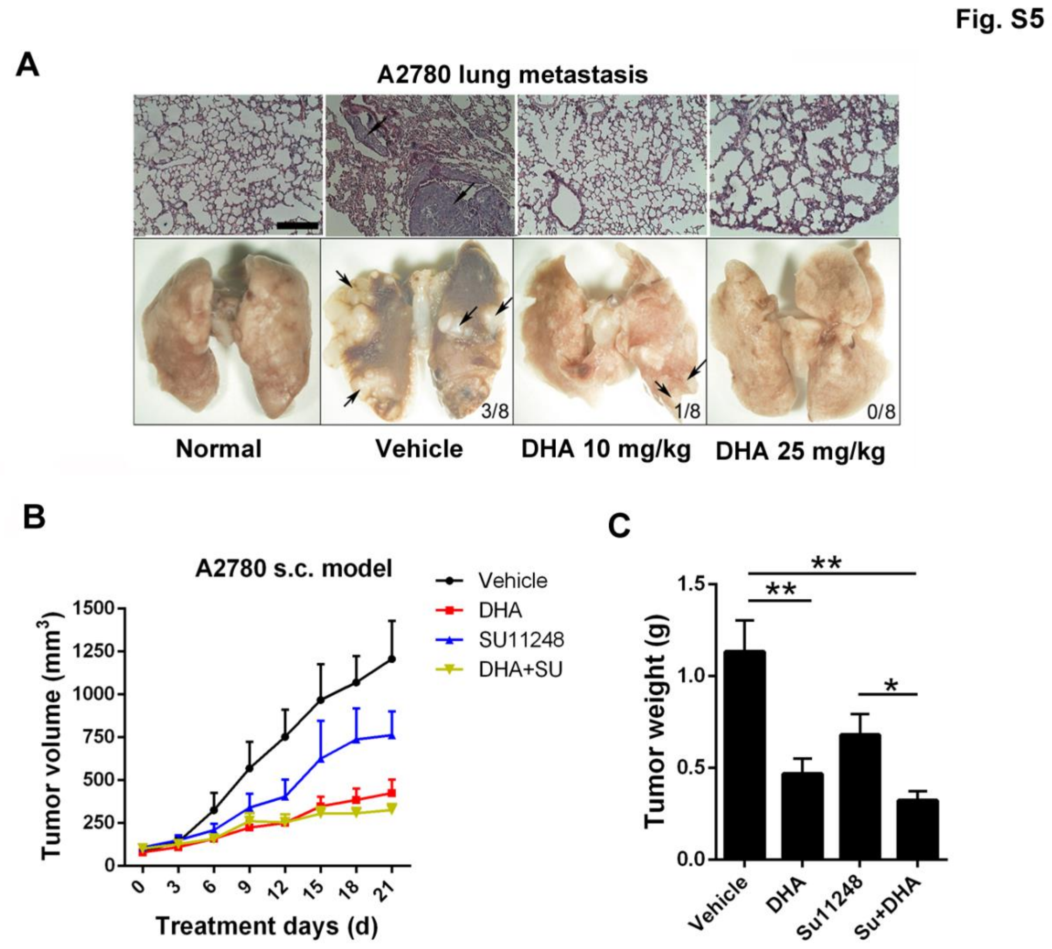

Figure S5 DHA inhibits ovarian cancer cell growth and sensitizes it to PDGFR inhibitor sunitinib in vivo.

(A) The gross and microscopic histological analyses of the lung metastases of ovarian cancer cells in the control and DHA treated mice. The arrowheads indicate metastases. Scale bar, 200 μm. **(B)** A2780 xenografts were treated with DHA 30mg/kg/day (i.p.), sunitinib 30mg/kg/day (intragastric administeration, i.g.), or the combination of the two. The subcutaneous tumor growth were measured (means ± SEM，n=5). **(C)** The subcutaneous tumors were harvested and weighed. Data are shown as means ± SEM (*P < 0.05, **P < 0.05).

Figure S6

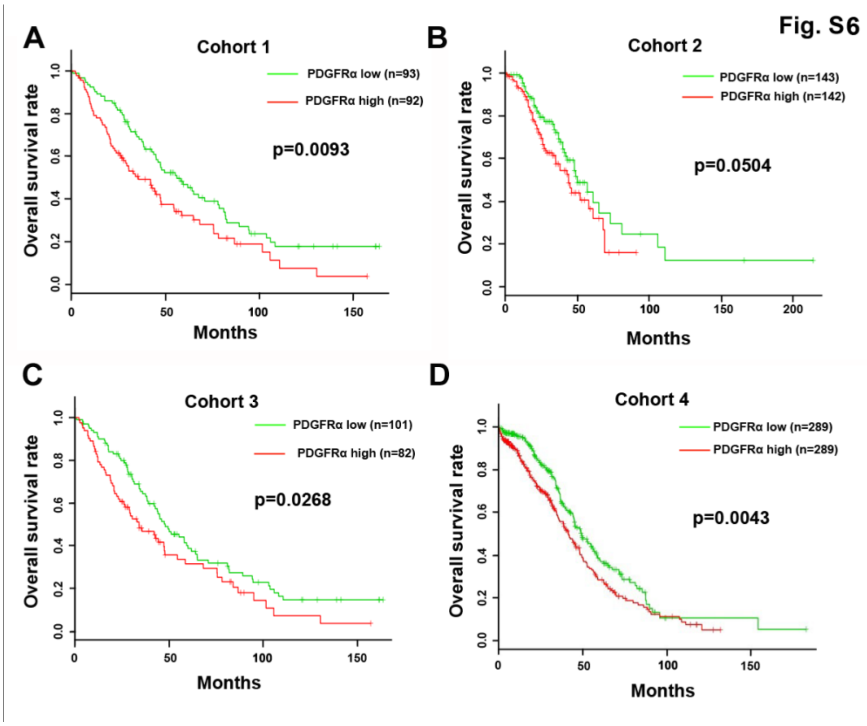

Figure S6 For four cohorts, there is an inverse association between PDGFRα expression and patients overall survival.

(A-D) Ovarian patients with high expression of PDGFRα (red curve) showed shorter overall survival times than those with low expression (green curve) across four datasets (all P valves < 0.05, log-rank test). The dataset of Cohort 1 (GSE 26712) and Cohort 2 (GSE9891) were from NCBI GEO database. The Cohort 3 and Cohort 4 were from TCGA database. Vertical hash marks indicate points of censored data.

Figure S7

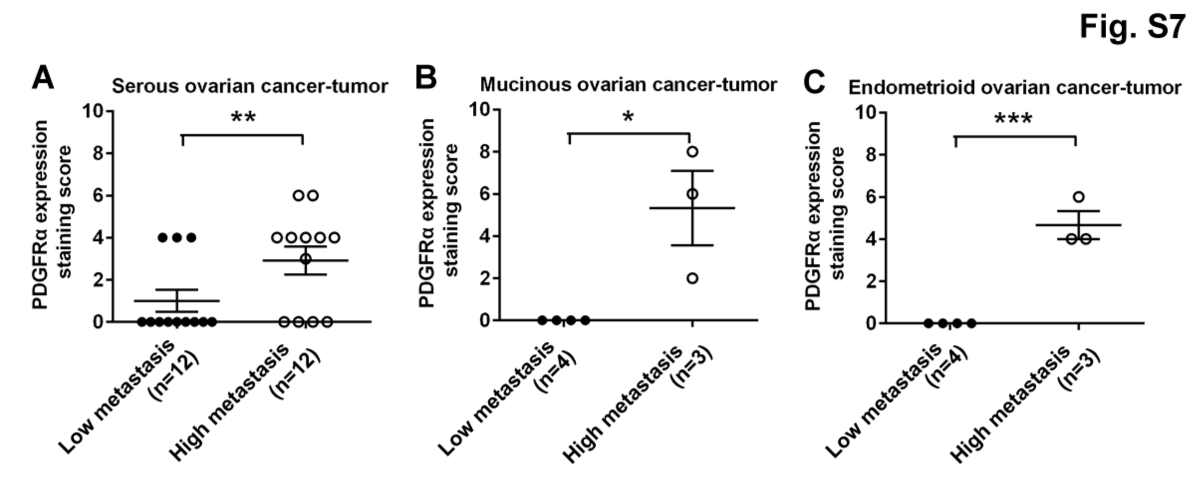

Figure S7 The PDGFRα expression is elevated in ovarian cancer tissues from patients with high metastatsis.

Based on the metastasis status, 45 ovarian cancer patients were divided into a low metastasis group (including 12 serous, 4 mucinous and 4 endometrioid) and a high metastasis group (including 12 serous, 3 mucinous and 3 endometrioid). The immunostaining intensity of the PDGFRα expression was scored in serous **(A)**, mucinous **(B)** and endometrioid **(C)** ovarian tumor cells from patients with low or high metastasis (means ± SEM; $^{*}P < 0.05$. $^{**}P < 0.01$, $^{***}P < 0.001$).

Supplementary tables:

Table S1. The membrane receptor proteins differentially expressed after DHA exposure.

UniProtKB accession number	Protein name	Theoretical molecular mass (kDa)/ p*I*	Sequence coverage (%)	Peptide count	Unique peptide count	Fold difference (Mean ± SD) DHA-treated/Control 24 h
P16234	Platelet-derived growth factor receptor alpha	122.7/5.04	10.8	4	2	0.499±0.007
P09619	Platelet-derived growth factor receptor beta	120.6/4.87	20.9	3	2	0.527±0.021
P00533	Epidermal growth factor receptor (EGFR)	134.3/6.17	10.8	3	2	0.447±0.035
P17948	Vascular endothelial growth factor receptor 1 (VGFR1)	150.8/8.72	26.4	8	3	0.460±0.044
P35968	Vascular endothelial growth factor receptor 2 (VGFR2)	151.5/5.57	19.5	20	7	0.465±0.013
P35916	Vascular endothelial growth factor receptor 3 (VGFR3)	152.8/5.87	12.2	29	3	0.431±0.020
O60603	Toll-like receptor 2	87.7/6.14	38.8	8	6	0.462±0.029
O00206	Toll-like receptor 4	95.7/5.78	14.5	4	3	0.468±0.005
Q9NR96	Toll-like receptor 9	116/8.5	20.6	8	3	0.471±0.052
P05556	Integrin beta-1	88.4/5.39	10.2	4	3	0.487±0.012
P05106	Integrin beta-3	87.1/4.97	15.8	7	3	0.491±0.004
P03372	Estrogen receptor (ER-alpha)	66.2/8.53	15.5	5	2	0.492±0.002
P10275	Androgen receptor (AR)	98.9/6.05	20.8	3	2	0.477±0.038
O00264	Membrane-associated progesterone receptor component 1 (PGRMC1)	21.7/4.70	15.9	4	2	0.473±0.035
Q93084	Sarcoplasmic/endoplasmic reticulum calcium ATPase 3 (SERCA3)	113.9/5.41	24.2	14	3	0.490±0.004
Q14994	Constitutive androstane receptor	39.9/8.54	29.8	6	5	1.983±0.062
O14763	Death receptor 5 (DR5)	42.2/4.97	25.2	8	4	2.010±0.007
P25942	Tumor necrosis factor receptor superfamily member 5 (CD40)	30.6/5.29	38.9	20	5	2.124±0.106
P29965	CD40 ligand (CD40L)	29.3/8.53	23.9	8	3	2.058±0.080
P02786	Transferrin receptor protein 1	84.9/6.18	13.3	2	2	2.026±0.039
O75469	Orphan nuclear receptor PXR	49.8/8.70	20.1	7	6	2.013±0.020

Table S2.The metastasis- and EMT-related proteins that were differentially expressed after DHA exposure.

UniProtKB accession number	Protein name	Theoretical molecular mass (kDa)/ p*I*	Sequence coverage (%)	Peptide count	Unique peptide count	Fold difference (Mean ± SD) DHA-treated/Control
						24 h
P12830	E-cadherin	97.5/4.23	26.5	12	4	2.028±0.035
P35221	Catenin alpha-1	100.1/6.29	17.1	29	10	2.002±0.082
B4DE16	Beta-catenin-like protein 1	61.9/5.08	15.2	3	3	2.018±0.006
P19022	N-cadherin	82/4.43	17.5	7	2	0.473±0.024
P08670	Vimentin	53.6/5.12	70.1	73	31	0.461±0.023
O43623	Neural crest transcription factor Slug	30/9.01	14.5	4	2	0.471±0.002
Q15672	Twist-related protein 1	20.9/9.48	16.5	4	2	0.431±0.017
P37275	Zinc finger E-box-binding homeobox 1(ZEB1)	124.1/4.87	13.7	4	2	0.418±0.007
P35222	Beta-catenin	85.5/5.53	37.7	13	5	0.434±0.009
P08253	Matrix metalloproteinase-2 (MMP2)	73.9/5.02	15.2	7	3	0.403±0.026
P09237	Matrix metalloproteinase-7 (MMP7)	66.6/5.44	25.2	8	5	0.483±0.010
P14780	Matrix metalloproteinase-9 (MMP9)	78.5/7.15	11.9	14	5	0.488±0.008
O94776	Metastasis-associated protein MTA2	74.9/9.66	15.8	10	2	0.446±0.038

Table S3. The clinicopathological characteristics of the patients and the expression of PDGFRα in ovarian cancer.

Characteristic	No. of cases	
	low grade (I-Ⅱ)	high grade (Ⅲ-Ⅳ)
**Ovarian carcinoma**	21	24
**Age at diagnosis, y**		
mean ± SD	54.6 ±7.6	53.6 ±7.0
**Type of differentiation**		
Low	0	24
Moderate	12	0
High	9	0
**Histotype (diagnosis)**		
Serous	12	12
Mucinous	4	3
Endometrioid	4	3
Other	1	6
**Metastasis (at surgery)**		
Non/mild-metastatic*	21	0
Extensive metastasis **	0	24
**PDGFRα staining score**		
Tumor: < 4	19	9
Tumor: ≥4	2	15

* Mild metastasis: Some cases were found with small metastases near the ovary or ipsilateral oviduct at the time of surgery.

**Extensive metastasis: The cases were diagnosed with extensive lymphatic and abdominal metastasis to multiple organs, including the contralateral ovary, bilateral oviduct, omentum, peritoneum, uterus and appendix caecalis.

Table S4. The primary antibodies used in this study.

Antigen	Source	Vendor
p-PDGFRα (Tyr754)	rabbit	Cell signaling technology
PDGFRα	rabbit	Cell signaling technology
p-PDGFRβ (Tyr751)	rabbit	Cell signaling technology
PDGFRβ	rabbit	Cell signaling technology
E-cadherin	mouse	BD Bioscience
Vimentin	mouse	Sigma
N-cadherin	mouse	Millipore/upstate
p-AKT(Ser473)	rabbit	Cell signaling technology
AKT	rabbit	Cell signaling technology
p-ERK1/2(Thr202/Tyr204)	rabbit	Cell signaling technology
ERK1/2	rabbit	Cell signaling technology
p-P38 MAPK(Thr180/Tyr182)	mouse	Cell signaling technology
P38 MAPK	rabbit	Cell signaling technology
p-SAPK/JNK(Thr183/Tyr185)	mouse	Cell signaling technology
SAPK/JNK	rabbit	Cell signaling technology
Twist	rabbit	Santa cruz
Slug	mouse	Santa cruz
β-catenin	mouse	Cell signaling technology
Cyclin D1	mouse	Santa cruz
GAPDH	mouse	Santa cruz
β-actin	mouse	Sigma

Table S5. The primers used in this study.

Target	Primer sequence (5’-3’)
PDGFRα	Sense: TGTCCTGGTTGTCATTTGGA
	Antisense: CTTCAACCACCTTCCCAAAC
PDGFRβ	Sense: GTGCTCACCATCATCTCCCT
	Antisense: ACTCAATCACCTTCCATCGG
E-cadherin	Sense: GACAACAAGCCCGAATT
	Antisense: GGAAACTCTCTCGGTCCA
Vimentin	Sense: TTGAAAATCCAGCGTGGACA
	Antisense: GATTCCACTTTGCGTTCAAGGT
Slug	Sense: ATGAGGAATCTGGCTGCTGT
	Antisense: CAGGAGAAAATGCCTTTGGA
Twist	Sense: GTCCGCAGTCTTACGAGGAG
	Antisense: CCAGCTTGAGGGTCTGAATC
Snail	Sense: GGTTCTTCTGCGCTACTGCT
	Antisense: TAGGGCTGCTGGAAGGTAAA
β-catenin	Sense: TTGAAAATCCAGCGTGGACA
	Antisense: TCGAGTCATTGCATACTGTC
c-myc	Sense: GCCACGTCTCCACACATCAG
	Antisense: TGGTGCATTTTCGGTTGTTG
18S	Sense: CAGCCACCCGAGATTGAGCA
	Antisense: TAGTAGCGACGGGCGGTGTG
